# Remission of anorexia nervosa after thyroidectomy: A report of two cases with Graves' disease and anorexia nervosa

**DOI:** 10.1186/1756-6614-4-17

**Published:** 2011-12-01

**Authors:** Hitoshi Noguchi, Tsukasa Murakami, Shinya Uchino, Hiroto Yamashita, Shiro Noguchi

**Affiliations:** 1 Noguchi Thyroid Clinic and Hospital Foundation, 6-33 Noguchi-Nakamachi, Beppu, Oita, 874-0932 Japan

**Keywords:** thyroid, anorexia nervosa, thyroidectomy, avian migration

## Abstract

We report two patients with anorexia nervosa and Graves' disease who received subtotal thyroidectomy for Graves' disease and concomitantly experienced remission from anorexia nervosa. Both were young women (aged 20 and 26) at the time of surgery. Both had well controlled thyroid function and eating behavior at the time of surgery. Both were followed for over five years without relapse of anorexia nervosa or hyperthyroidism. These cases suggest the existence of an endocrine factor originating from the thyroid gland that is involved in the pathogenesis of anorexia nervosa. Since patients of thyroidectomy can remain in good health with supplement of thyroxine alone, it can be hypothesized that this anorexigenic endocrine factor is an evolutionary relic not necessary for the normal function of humans and does not have physiological effects unless secreted beyond normal levels. Given that, it implies the existence of a creature in the animal kingdom for which such an anorexigenic hormone is essential for survival. Migrating birds eat beyond their caloric expenditure before migration and become anorexic for the duration of their flight. It is also known that their thyroid function is elevated during migration. The normal physiology of migration is a complex mechanism involving the hypothalamic, pituitary, thyroid, adrenal and reproductive hormones. The mechanism of disease, however, can be simpler. A review of the literature is presented that suggest a heretofore unreported thyroid hormone, which is involved in the regulation of migration behavior, may be the responsible factor behind anorexia nervosa.

## Background

Anorexia nervosa is a potentially fatal eating disorder with a strong psychological component. There is still some disagreement over whether the disease is primarily psychological with physical changes as a result of malnutrition, or a physical disease that results in behavioral changes [1,2]. It has recently been demonstrated that peptide YY (PYY), glucagons-like peptide 1 (GLP-1) and ghrelin behave in opposite ways between patients of anorexia nervosa and constitutionally thin subjects [3], suggesting that endocrine changes precede malnutrition in anorexia nervosa. However, the actual endocrine mechanism behind anorexia nervosa is not known. Here we present two cases of young women with Graves' disease and anorexia nervosa who underwent surgery for Graves' disease and concomitantly experienced remission of anorexia nervosa. These cases suggest that an anorexigenic endocrine factor is secreted from the thyroid gland. A review of the literature is discussed.

## Case Presentation

### Case 1

20 year old female (upon admission). Diagnosed of Graves' disease the previous year but presented allergic urticaria to both propylthiouracil and methimazole. Thyroid function was controlled with potassium iodide until surgery. She had a history of anorexia bulimia and was hospitalized for cognitive behavioral therapy at age 18. Her eating behavior was stable as well as her thyroid function at the time of surgery at age 20. There is no record of the use of SSRIs in the treatment of anorexia nervosa. Her height was 154.2 cm and body weight was 53 kg (BMI 22.3) at the time of surgery. Post operatively she was placed on 50 mcg of levothyroxine daily which maintained her thyroid function within normal levels. There was no relapse of her anorexia bulimia and her body weight was maintained over the next five years.

### Case 2

26 year old female (upon admission). Diagnosed of Graves' disease at age 26 but presented with liver dysfunction with methimazole and mild neutropenia with propylthiouracil. Thyroid function was controlled with potassium iodide until surgery. She had a history of anorexia and bulimia since age 19. Her thyroid function and eating behavior were both stable at the time of surgery. There is no record of the use of SSRIs in the treatment of anorexia nervosa. Her height was 158.5 cm and her weight was 55.0 kg (BMI 21.9) at the time of surgery. Post operatively she was placed on 75 mcg of levothyroxine daily which maintained her thyroid function within normal levels. There was no relapse of her anorexia bulimia and her body weight increased slightly and gradually over the next seven years (to BMI 23.8).

## Conclusions

The weight of these cases is limited by the fact that the psychological case files are not available for this report. However, both cases have documented records of anorexia bulimia and body weight fluctuations spanning at least 10 Kg. Both cases have experienced prolonged remission from anorexia bulimia after subtotal thyroidectomy.

These observations suggest that there may be an endocrine substance that is involved in the pathogenesis of anorexia nervosa. Since patients who had undergone total thyroidectomy are known to remain in perfect health with supplementation of only levothyroxine, it is believed that thyroxine is the only biologically essential hormone secreted by the thyroid gland. However, it is also known that hormones such as calcitonin are secreted by the thyroid gland but does not need to be supplemented in thyroidectomized humans. Although calcitonin does have physiological effects on humans, its absence does not usually produce significant physiological changes. Likewise, the anorexigenic thyroid hormone may also be something that can have physiological effect when over secreted, but its absence is not significant in humans. Such a hormone may be an evolutionary relic, important for the survival of our evolutionary ancestors but no longer necessary in humans.

Extending the hypothesis further, if the anorexigenic thyroid hormone is an evolutionary relic, it is possible that there is a creature in the animal kingdom for which the hormone is vital for survival. Migratory birds are a possible candidate. Birds eat beyond their caloric expenditure before migration and become anorexic during flight. They are hyperactive while they are anorexic [4]. The anorexigenic hormone may be involved in the control of feeding behavior of migrating birds and/or animals.

Experiments aimed at inducing zugunruhe (behavior indicating migration) with birds injected with thyroid extract and birds injected with thyroxine tend to contradict each other, especially at large doses [5]. This suggests the existence of an active substance in the thyroid extract other than thyroxine.

Anorexia nervosa is more often found in industrialized countries, which may contradict the theory that it is an endocrine disease. This may partly be the result of diagnostic bias from the difference in the background prevalence of simple malnutrition in industrialized and non-industrialized countries, as well as the difference in the likelihood that anorexia nervosa is accurately diagnosed. But if a hormone involved in the control of migration is responsible for the pathogenesis of anorexia nervosa, it may also stem from the fact that residents of industrialized countries, where people tend to be awake late into the night under artificial light, are more likely to be desensitized to photoperiodic signals than the residents of non-industrialized countries, where the lights go out early. Photoperiodic signals are a known cue for migration behavior [6].

Likewise, anorexia nervosa is sometimes successfully treated by cognitive behavioral therapy. This may also seem to contradict the hypothesis that anorexia nervosa is an endocrine disease, but a regimen that enforces regular sleeping schedules can help restore sensitivity to photoperiodic signals.

Other factors more commonly found in industrialized countries, such as nutritional, pharmacological, electro-magnetic, thermal or social stimuli, may mimic migratory cues intermittently and confound the endocrine responses. Most cognitive behavioral therapies tend to isolate the patient from such stimuli which may be the reason they are sometimes effective on mild to moderate cases.

Although the normal physiology of migration is a complex mechanism involving a large number of hormones working in unison, and the normal physiology of body weight regulation is no less complicated, the physiology of a disease is often simpler. It takes the hyper-physiological dose of just one hormone to cause a disease. Identifying the hormone may help identify the cure.

The above theory is so far only hypothetical and further studies must be performed to evaluate its validity. However, these cases may represent a substantial clue in the study anorexia nervosa.

## Consent

The patients have given written consent for the information included above to be published.

## Competing interests

The authors declare that they have no competing interests.

## Authors' contributions

HN has followed, analyzed and interviewed the patients, formulated the theory and drafted the manuscript. TM diagnosed the Graves' disease and aided in the treatment. SU performed the surgery. HY performed the pathology. SN authorized the report. All authors have read and approved the final manuscript.
